# A novel COL6A2 mutation causing late-onset limb-girdle muscular dystrophy

**DOI:** 10.1007/s00415-019-09307-y

**Published:** 2019-04-08

**Authors:** Manu Jokela, Sara Lehtinen, Johanna Palmio, Anna-Maija Saukkonen, Sanna Huovinen, Anna Vihola, Bjarne Udd

**Affiliations:** 1 Neuromuscular Research Center, Department of Neurology, Tampere University and University Hospital, Tampere, Finland; 2 Division of Clinical Neurosciences, Turku University Hospital, University of Turku, Kiinamyllynkatu 4-8, 20520 Turku, Finland; 3 Neuromuscular Research Center, Fimlab Laboratories, Tampere University and University Hospital, Tampere, Finland; 4 Department of Neurology, Northern Karelia Central Hospital, Joensuu, Finland; 5 Department of Pathology, Fimlab Laboratories, Tampere University Hospital, Tampere, Finland; 6 Department of Medical Genetics, Folkhälsan Institute of Genetics, Haartman Institute, University of Helsinki, Helsinki, Finland; 7 Department of Neurology, Vasa Central Hospital, Vasa, Finland

**Keywords:** Myopathy, Collagenopathy, Limb-girdle muscular dystrophy, COL6A2

## Abstract

Limb-girdle muscular dystrophies (LGMD) are genetic disorders characterized by weakness of predominantly proximal limb and trunk muscles due to progressive loss of muscle tissue. Collagen VI-related muscular dystrophies usually display more generalized muscle involvement combined with contractures and/or hyperlaxity of distal finger joints. LGMD-like phenotype of collagenopathy has only rarely been described and as reported is usually of childhood onset. We identified a Finnish family with *COL6A2*-related LGMD with autosomal dominant inheritance and very late onset at 40–60 years of age. Since the mutation was previously unreported, the pathognomonic findings on muscle MRI were the decisive clue for the correct diagnosis.

## Introduction

Limb-girdle muscular dystrophies (LGMD) are genetic disorders characterized by progressive weakness and loss of muscle tissue predominantly affecting proximal limb muscles. The degeneration of muscles is detectable both on muscle MRI and biopsy, which results in end-stage pathology in the severely affected muscles. LGMDs are further subdivided into autosomal dominant and autosomal recessive forms. The growing number and increasing heterogeneity of different LGMD forms reported over the years prompted a recent European Neuromuscular Center (ENMC) workshop to suggest revisions for the definition and classification of LGMD [1].

Muscle collagenopathies are caused by mutations in *COL6A1*, *COL6A2* or *COL6A3* genes*,* which encode different alpha-subunits of fibroblast-secreted collagen VI fibrils deposited in the extracellular matrix. Collagen VI is required for the physical integrity and cohesion of muscle tissue as well as for signaling functions. The phenotype of collagenopathies usually includes a combination of contractures with progressive muscle weakness and wasting of variable severity [2]. An LGMD-like phenotype without significant contractures has been reported only rarely [3].

We describe a Finnish family with *COL6A2*-related dominant LGMD and very late onset (40–60 years). An unreported, unique mutation in the *COL6A2* gene (c.2107A>C p.T703P, according to the transcript NM_001849) was identified in the proband using our targeted next-generation sequencing assay of myopathy-associated genes (MyoCap) [4]. Sanger sequencing confirmed correct segregation of the detected mutation in three other family members.

## Materials and methods

### Patients

Four members of a Finnish family with late-onset, autosomal dominant myopathy underwent clinical and laboratory, muscle imaging, histopathological and molecular genetics studies to identify the causative genetic defect. All patients provided informed consent for the examinations and the study was approved by the Tampere University Hospital ethics board.

### DNA sequencing

DNA samples of the proband (II-2) and one of his affected sister’s (II-1) were studied by targeted next-generation sequencing. The exons of 363 myopathy-associated genes were targeted using our custom MYOcap assay (Roche Nimblegen, Madison, WI, USA). The list of targeted genes is available upon request. Nimblegen SeqCap EZ Choice Library protocol was used for capture and target enrichment. The enriched library was sequenced by Illumina HiSeq4000 Sequencher to 75 bp paired-end read length. The capture, enrichment and next-generation sequencing were performed at Oxford Genomics Centre (OGC at Wellcome Trust Centre for Human Genetics, Oxford, UK). The read/raw data were analyzed using an in-house developed pipeline [4].

Sanger sequencing of exon 26 of *COL6A2* gene was used to analyze DNA samples of family members II-3 and II-4. The exon of interest was amplified by PCR (2× PCR Master Mix; ThermoFisher Scientific, Waltham, MA, USA) and sequenced using Big-Dye Terminator v3.1 Kit on an ABI3130×l automatic Genetic Analyzer (Applied Biosystems, Foster City, CA, USA). Primer sequences are available upon request. Sequence analysis was performed with Sequencher 5.1 software (Gene Codes Corporation, Ann Arbor, MI).

### Muscle MRI

MRI scans with axial sections of the lower limb muscles and using T1-weighted and short TI inversion recovery sequences were evaluated in four family members (II-1, II-2, II-3, II-4).

### Muscle histopathology and immunofluorescence studies

Muscle biopsies were analyzed in patients II-1, II-2 and II-4, including hematoxylin and eosin (H&E), Gomöri trichrome, NADH-TR, COX-SDH and neonatal myosin heavy chain (MyHC) stains. MyHC slow and MyHC fast double staining was also assessed. For the immunofluorescence studies, the primary antibodies used for immunofluorescent double staining were mouse monoclonal anti-collagen VI, clone VI-26 (Merck MAB3303) and rat monoclonal anti-merosin/LAMA2, clone 4H8-2 (Abcam ab11576). Muscle biopsies [patients II-1 and II-2; healthy control free of muscle disease; and disease control, a patient with early-onset collagenopathy (*COL6A1* c.1056+1G>A p.G335_D352del)] were snap-frozen in liquid nitrogen-cooled isopentane, and 6 µm muscle cryosections were prepared on SuperFrost + sides. The sections were fixed with 4% PFA for 15 min, and permeabilized using 0.05% Triton X-100 in PBS for 10 min. After blocking with 2% BSA for 30 min, the pooled primary antibody mix was added and incubated at + 8 °C overnight. The following day, the slides were washed with PBS, and secondary antibody incubation was performed using Alexa-546 and Alexa-488 conjugated antibodies, for 1 h at room temperature. An Axioplan 2 epifluorescence microscope (Carl Zeiss Microscopy GmbH) was used for imaging.

## Results

**Fig. 1:**
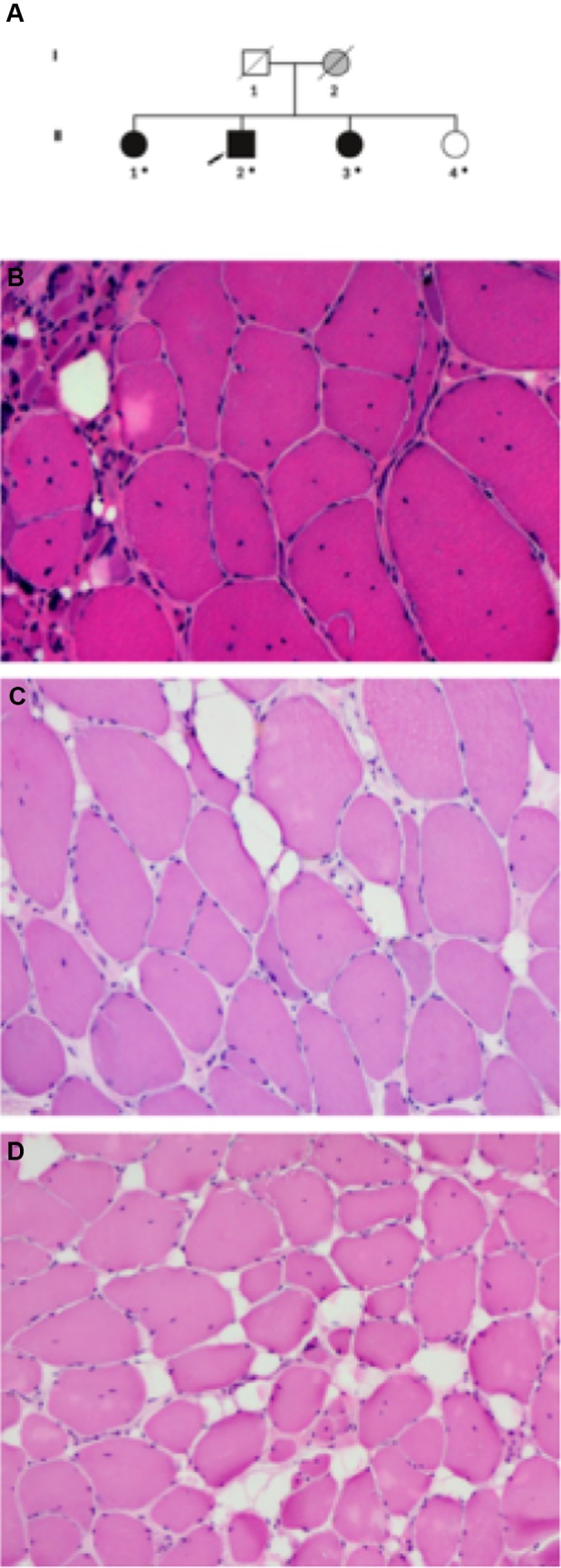
**a** Pedigree of the family. Solid black symbols represent clinically affected individuals. Individual I-2 labeled solid gray has not been clinically evaluated but was reportedly affected. DNA sample was available from individuals marked with a dot. **b**–**d** Pathological changes observed in tibialis anterior muscle biopsies (Haematoxylin andEosin stain). Muscle biopsy from the proband (II-2) shows dystrophic features including many internalized nuclei, fiber size variation, fiber splitting and increase of connective and adipose tissue. Small group atrophy signifying neurogenic involvement is also present, probably related to an additional radiculopathy in this patient. Biopsies obtained from the proband’s sisters (II-1, II-3; **c**, **d**, respectively) showed fiber atrophy, internal nuclei and endomysial fibrofatty degeneration

The proband (II-2) first noticed proximal weakness in the lower limbs when lifting heavy objects or walking up stairs at the age of 59–60 years. Previously he had been athletic and in his forties he was still able to ski for 10 km. After age 62, he had also experienced weakness in proximal upper limbs. On examination at age 66, he had proximal weakness in the upper and lower limbs of MRC grade 3–4, slight scapular winging and distal weakness in the lower limbs (Table 1). EMG/NCS and muscle biopsy (Fig. 1, Table 1) showed abnormalities compatible with dystrophy, but also signs of neurogenic involvement most likely related to previous L4 radiculopathies.

**Table 1:** Clinical findings in affected family members

Patient	Sex/age	Age at onset	First symptoms	Muscle weakness	Walking aids	Heart involvement	CK level	EMG	Imaging	Muscle biopsy
II:1	F/70	55	Difficulties climbing stairs and rising from a squat	Proximal LL weakness: knee ext 4/3, other 4,5 Tight heel cords No UL weakness Mild scapular winging	No	No	*N*–1.5 × UNL	Normal	Tigroid pattern of fatty replacement	Tibialis anterior: fiber atrophy, mild endomysial fibrosis and fatty degeneration, internal nuclei, multiminicores, few rimmed vacuoles
II:2	M/68	59–60	Difficulties climbing stairs, squatting Proximal UL weakness	Scapular winging UL: abduction 4, triceps 4 LL: hip extension 3-, knee flexion/extension 4, ankle dorsiflexion 2/3 right/left	Canes for longer distances, AFO on right side	No	1.5 × UNL	Nonspecific abnormalities consistent with myopathic and/or neurogenic origin	Tigroid pattern of fatty replacement	Vastus lateralis: absent type I fibers, lobulation Tibialis anterior: severe dystrophic changes and neurogenic features, few rimmed vacuoles
II:3	F/62	40	Difficulties climbing stairs and squatting, walking on heels	Proximal LL weakness, limited ankle dorsiflexion	No	No	Elevated	Myopathy	Tigroid pattern of fatty replacement	Tibialis anterior: fiber atrophy, mild endomysial fibrosis and fatty degeneration, internal nuclei

*AFO* ankle foot orthosis, *LL* lower limbs, *F* female, *M* male*, UL* upper limbs, *TA* tibialis anterior, *UNL* upper normal limit

**Fig. 2:**
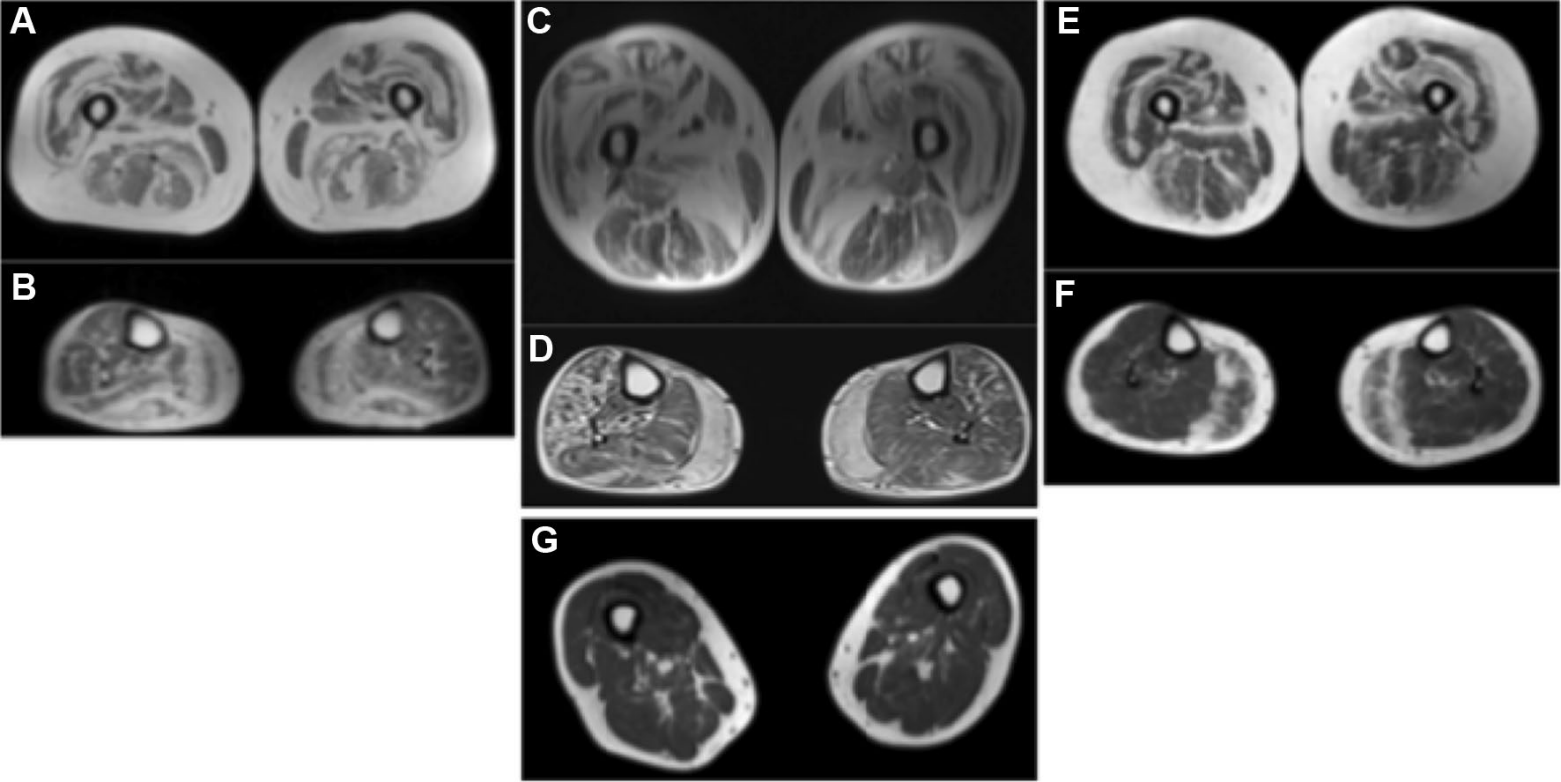
Lower limb magnetic resonance imaging shows characteristic fatty degeneration in the center of the rectus femoris and a layered, "tigroid" pattern in the vastus lateralis of patients II-1 (**a**), II-2 (**c**), II-3 (**e**), while thigh MRI was normal in the unaffected sibling II-4 (**g**) at 60 years of age. There is severe fatty replacement of the gastrocnemius medialis in all patients (**b**, **d**, **f**)

His two sisters (II-1, II-3) also had a similar phenotype characterized by proximal muscle weakness from ages 40 and 50 years onwards, respectively. Lower limb muscle MRI showed layers of fatty replacement in a “tigroid” pattern (Fig. 2a, c and e). Muscle biopsies showed myopathic features including fiber atrophy, increased internal nuclei and mild fibrofatty tissue replacement (Table 1, Fig. 1). None of the patients had significant joint contractures, although range of motion in ankle dorsiflexion was limited due to mildly tight heel cords.

The patients were confirmed to carry a heterozygous c.2107A>C p.T703P mutation in the *COL6A2* gene (pedigree in Fig. 1). Targeted next-generation sequencing specified seven rare variants (MAF < 0.01) shared between proband and his affected sister II-1. Of these, only c.2107A>C in *COL6A2* gene was unique and, therefore, considered likely pathogenic. This was supported by in silico pathogenicity prediction programs (Polyphen, Sift, Provean, Mutation Taster), which all predicted the variant to be damaging. Missense or synonymous variants in *OBSCN, PLEC, PLEKHG4, NEB, ITGA7* were excluded on the basis that they were too common (MAF > 0.001) for a fully penetrant dominant mutation and because they were classified as benign/tolerated using in silico pathogenicity assessment tools. Furthermore, one of the variants was in a gene that is not expressed in skeletal muscle (*MYLK3*).The same *COL6A2* mutation was detected by Sanger sequencing also in the affected sister II-3. One of the sisters (II-4) had minor myalgic complaints at age 60 years and was, therefore, also studied by muscle MRI, which showed normal findings (Fig. 2). She was found not to carry the *COL6A2* variant that was detected in the affected family members.

**Fig. 3:**
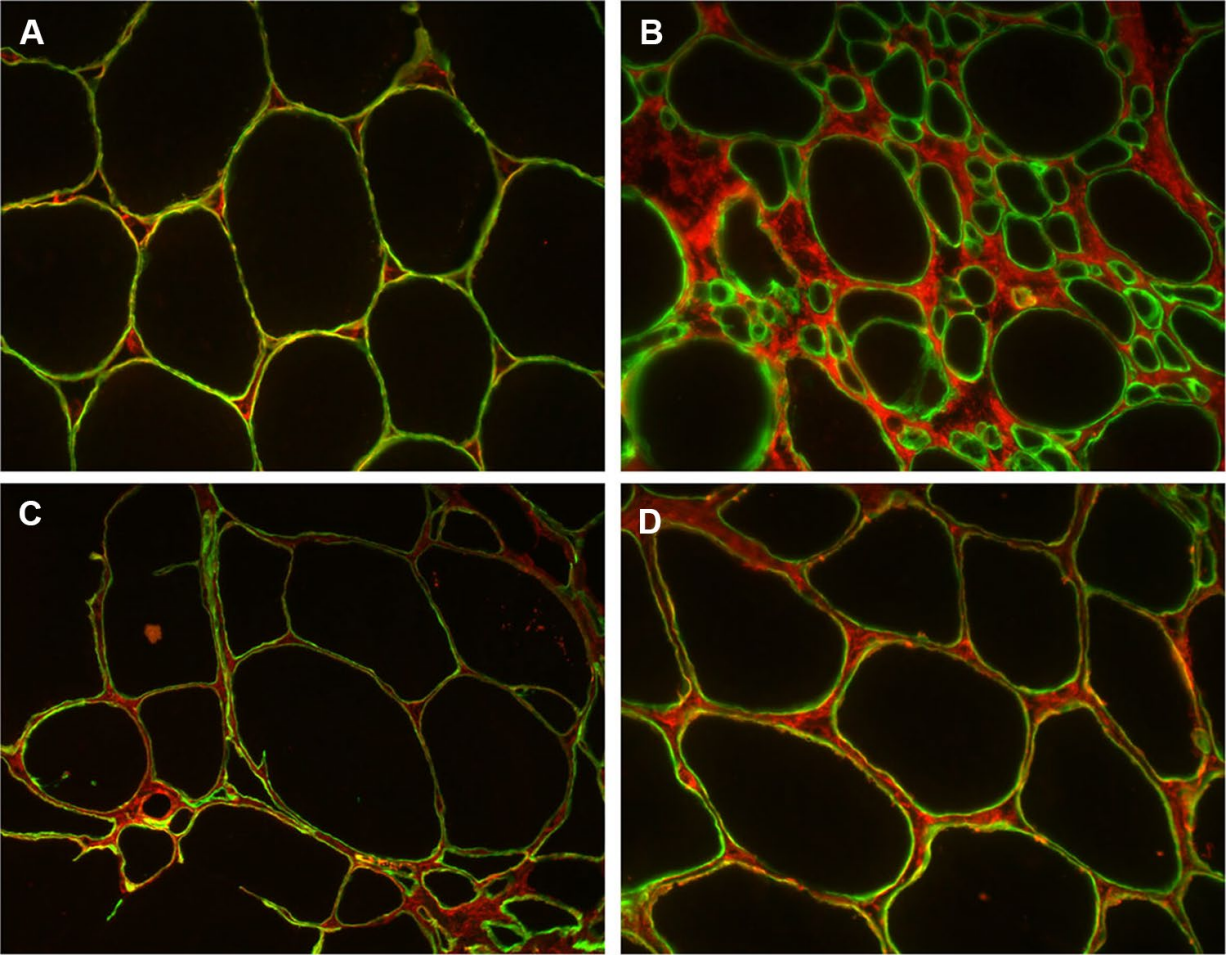
Double staining using collagen VI (red) and merosin/LAMA2 (green) antibodies in the normal control muscle (**a**) shows co-localization of the two labels, visualized as yellow labeling on the muscle fiber basal lamina. In a patient with early-onset severe collagenopathy (COL6A1), collagen (red) is largely detached from the basal lamina, resulting in green merosin labeling (**b**). The staining pattern in the proband’s II-2 (**c**) and his sister’s II-1 (**d**) muscle biopsies is not clearly abnormal, even though the interstitial space is enlarged showing collagen expression

We did not observe clear abnormality in the immunofluorescent double staining of COLVI and merosin/LAMA2 in the muscle biopsies from patients II-1 and II-2, indicating that there is at least partial co-localization of these proteins, visualized as mostly yellow overlapping labeling in the muscle fiber basal lamina (Fig. 3c, d).

## Discussion

We describe a family with three patients having an LGMD-like phenotype of collagenopathy with atypical late onset. Symptoms in Bethlem myopathy (OMIM #158,810) usually start in childhood with the majority of patients requiring mobility aids after 50 years of age [5].

Although distal lower limb weakness was present at the later disease stages in our patients, the initial symptoms in all our patients were due to proximal muscle weakness. A unique variant in *COL6A2* (c. 2107 A>C p.T703P) was found to fully segregate with the phenotype in this family. Pathogenicity of the mutation is supported by the unusual and highly characteristic muscle MRI pattern, which is almost exclusively seen in collagenopathies [2], although it has also been rarely reported in calpainopathy [6]. Double immunofluorescence of collagen VI and merosin did not show obvious abnormalities, which is in line with previous reports in milder dominantly inherited collagenopathies, such as Bethlem myopathy. The double staining IF method is more useful in severe collagenopathy, where COL VI is clearly detached from the myocyte basal lamina, and the co-localization of COL VI and merosin is lost [2].

Collagen VI-related disorders are autosomal dominant or recessive disorders with phenotypes ranging from severe congenital muscular dystrophy to milder, adult-onset forms and are usually accompanied by variable degrees of joint contractures [2]. Our patients did not have contractures or spinal deformities, although some degree of Achilles tendon tightness was present in all. However, this is a nonspecific finding and not very significant considering the age of the patients.

LGMD-type of collagenopathy without contractures has been reported only rarely and according to the new ENMC classification, would be labeled as LGMD D5 (dominant) or LGMD R22 (recessive). The family reported here meets the criteria for a late-onset form of LGMD D5, caused by the *COL6A2* mutation c. 2107 A>C p.T703P.

Although sporadic inclusion body myositis is the most common muscle disease in patients over 50 years of age, it is important to recognize that several genetic myopathies and dystrophies may also manifest at this age. Indeed, some of the most common LGMD forms in Finland, such as LGMD2L (anoctaminopathy) and *DNAJB6*-related LGMD1D, frequently become symptomatic only after 50 years of age [7]. Accurate family history may be especially difficult to obtain in these very late-onset patients, because of the many other age-related neuromuscular complaints in this age group. Similarly, the proband (II-2) of our family had signs of neurogenic disease in the lower limbs both on EMG and muscle biopsy, which might have been considered the cause of his symptoms, had the muscle MRI not been performed. Our study, therefore, shows that muscle MRI is a valuable tool to help sorting out different neuromuscular disorders also in elderly patients.
